# The Code of the Street Fights Back! Significant Associations with Arrest, Delinquency, and Violence Withstand Psychological Confounds

**DOI:** 10.3390/ijerph17072432

**Published:** 2020-04-03

**Authors:** Kyle A. Burgason, Matt DeLisi, Mark H. Heirigs, Abdi Kusow, Jacob H. Erickson, Michael G. Vaughn

**Affiliations:** 1 Department of Sociology and Criminal Justice, Iowa State University, Ames, IA 50011, USA; burgason@iastate.edu (K.A.B.); kusow@iastate.edu (A.K.); 2 Department of Sociology, University of Montana, Missoula, MT 59812, USA; mheirigs@iastate.edu; 3 Department of Criminal Justice & Criminology, Georgia Southern University, Statesboro, GA 30460, USA; jacobe@iastate.edu; 4 School of Social Work, Saint Louis University, Saint Louis, MO 63103, USA; michael.vaughn@slu.edu; 5 Graduate School of Social Welfare and College of Social Science, Yonsei University, Seoul 03722, Korea

**Keywords:** code of the street, delinquency, violence, temperament, psychopathy, criminological theory

## Abstract

Since Anderson’s now classic, *Code of the Street: Decency, Violence, and the Moral Life of the Inner City*, an increasing number of researchers have found a significant association between the code of the street and antisocial behavior. Less researched, however, is the relationship between the code of the street and cognate psychological factors. Building on the hypothesis that the code of the street is simply a reflection of elements of the population who exhibit antisocial traits, our aim in this study is to empirically test whether the observed association between the code of the street and antisocial behavior can withstand psychological confounds among a sample of institutionalized juvenile delinquents. Negative binomial regression models show that the code of the street remained a significant predictor of antisocial behavior despite the specification of psychopathy and temperamental traits and other controls. Moreover, as theorized, differential effects were found for African American delinquents compared to non-African American delinquents. We discuss theoretical and practical implications.

## 1. Introduction

Elijah Anderson’s [1,2] *Code of the Street: Decency, Violence, and the Moral Life of the Inner City* is among the most influential and acclaimed theoretical developments in criminology in recent decades, one that has promulgated to the broader culture. Anderson’s work tackles one of the most controversial empirical issues in American society, namely the high rate of interpersonal violence, homicide perpetration, and homicide victimization among inner city African American males. Multiple data sources indicate that homicide offending and victimization rates among African American males are exponentially higher than others in the U.S. population [3,4,5,6,7]. Since its publication, a plethora of investigators reported significant associations between the code of the street and diverse specifications of antisocial behavior among a variety of data sources, including the Mobile Youth Survey [8], National Youth Survey [9], Family and Community Health Study [10,11], Gang Resistance Education and Training (GREAT) program [12], and Seattle Neighborhoods and Crime Survey [13] within the United States in addition to data from the Netherlands [14] and United Kingdom [15,16]. Despite the diversity of these studies, the overall conclusion is clear: the more an individual advocates the code of the street, the greater his or her involvement in externalizing, antisocial, and violent behaviors.

Despite the popularity of Anderson’s thesis differentiating antisocial (“street”) and prosocial (“decent”) adaptations about cultural and structural isolation, it is also plausible that the code of the street is conflated with psychological factors that also relate to antisocial responses. As suggested by prior researchers [17,18,19,20,21], the code of the street is conceptually congruent with a variety of constructs including hostile attribution bias, antisocial personality disorder, temperamental deficits, and psychopathy. Unfortunately, this important conceptual point is largely overlooked in the criminological literature.

## 2. Theoretical Framework

Anderson’s formulations about inner city oppositional culture build on the work of Thrasher [22,23], who notes that origins of crimes are based on two generalizations; the behavior problems of childhood and adolescence, and the malfunction of social institutions in the crime producing areas. Concentrations of these and other damaging structural factors are found to weaken social control while socially isolating residents within their communities. The establishment of neighborhood cohesion and shared legitimate values is limited, resulting in the development of an oppositional culture that further perpetuates violent behavior [24,25].

Anderson’s [1] ethnographic research on structurally disorganized and segregated Philadelphia neighborhoods expands upon this by demonstrating how social isolation can suppress mainstream values and isolate residents from mainstream society. He argues that due to these structural changes in neighborhoods resulting in decreased employment opportunities and increased disadvantage, “the trust and perceptions of decency that once prevailed in the community are increasingly absent” (p. 145), and in their place a “code of the streets” has developed, which emphasizes toughness, risk-taking, and the use of violence to achieve status. As Anderson points out “violent solutions to problems in disadvantaged neighborhoods are an essential part of the local subculture, a means of defending one’s honor and winning respect from residents. These cultural codes appropriate aggressive responses toward individuals who show disrespect, a rationale allowing those who are inclined to aggression to precipitate violent encounters in an approved way” [1], (p. 33). Thus, the code of the street is a set of behavioral and attitudinal norms that influence and inform interactions among individuals in structurally disadvantaged neighborhoods [26].

Anderson suggests that the residents of socioeconomically disadvantaged inner city communities live by two basic cultural orientations: “decent” and “street”. His discussion centers on the role of the street culture in exacerbating levels of violence where individuals gain or lose respect based on their response to challenges. Responding with violence, often regardless of the outcome, earns respect or “juice”, which is often considered critical. While most residents of even the most socioeconomically disadvantaged and violent communities are “decent” and not strongly committed to the street orientation or the code of the street, all residents are cognizant of the behavioral norms it prescribes. Residents understand that abiding by these norms may reduce their risk of victimization and increase their odds of surviving a violent encounter [27,28,29]. Individuals prescribing to the street cultural orientation often campaign for respect or “juice”. This revolves around the presentation of self with the basic requirement of being able to display “a predisposition to violence, where the public bearing must send the unmistakable, if sometimes subtle, message that one is capable of violence, and possibly mayhem, when the situation requires it” [1], (p. 72). This presentation of self includes facial expressions, direct communication, gait, and the individuals’ physical appearance. Failure to respond to these challenges violates the code of the street and thus results in a loss of status and respect, which may have dire consequences. Once one’s honor and reputation are questioned, they stand to lose respect among peers and may become victimization targets. For individuals in this milieu, respect and honor often hinge on the how they perform on the streets during such violent interpersonal encounters.

## 3. The Code of the Street and Psychological Factors

Despite supportive research, there is also evidence suggesting that other factors influence antisocial outcomes among impoverished youth above and beyond the code of the street. In their study of over 2000 youth selected from the Georgia Department of Juvenile Justice, [30] found significant evidence of street code beliefs among delinquents; however, these beliefs existed among youth irrespective of their rearing environment, not exclusively in urban environments. This suggests that more universal antisocial features not specific to a street code are also driving violent responses to interpersonal affronts and disputes. In a qualitative study of young, African American males who were co-victims of homicide, [31] reported high prevalence of Posttraumatic Stress Disorder (PTSD) symptoms, including acute stressors (100% prevalence), intrusion/thought intrusion (46% prevalence), avoidance (46% prevalence), negative alterations in cognition and mood (65%), and alterations in arousal and reactivity/hypervigilance (68%). Although code of the street normative valuations were present in their data, their study reveals that exposure to acute levels of crime, disorder, and violence works to cultivate a psychological state where violence is often potentiated.

Less research attention focused on the interrelationship between the code of the street and cognate psychological factors that are themselves associated with antisocial behavior. In a provocative editorial, [18] hypothesized that the code of the street simply reflects elements of the population who exhibit an array of antisocial traits. According to this perspective, “decent” and “street” residents share the same socioeconomic status and racial status, yet engage in diametrically different behaviors, the former prosocial and the latter antisocial. DeLisi speculated that a variety of constructs, such as hostile attribution bias, low self-control, psychopathic features, and negative emotionality share important variance with the street code. Baron [32] analyzed data from 400 homeless youth in Canada to see how well the street code withstood controls for anger, self-centeredness, and nerve, which captured stress immunity in the face of potential victimization. Across multiple regression models, [32] found that the code of the street maintained significant associations with self-reported violent offending despite these and other controls. Moreover, interaction terms for the street code and anger, self-centeredness, and nerve were also significant. Finally, [32] reported moderation effects whereby standard deviation increases in anger, self-centeredness, and nerve were commensurately linked with greater effect sizes between the street code and violence. In other words, youth who adhere to the street code and who exhibit clinically elevated anger, self-centeredness, and nerve were most likely to be violent.

The salience of anger and self-centeredness clearly implicates self-control theory [33]. On that point, several studies exist. [34] analyzed national survey data and found that lower self-control predicted street code attitudes. Curiously, neither the street code nor self-control were associated with offending in multivariate models. Alternatively, [35] found that low self-control and street code adherence predicted offending after controlling for each in the same model. McNeeley, Meldrum, and Hoskin [36] studied the convergent validity between self-control and the street code among a student sample. They found that each dimension of the low self-control construct—impulsivity, risk-seeking, simple tasks, physical orientation, self-centeredness, and anger—was significantly associated with street code values. Additionally, two aggregate measures of self-control (the Grasmick scale and Tangney scale) were also predictive of street code values. Similarly, [37] found that low self-control predicted street code attitudes and that the street code maintained significant associations with self-reported violence and property offending, but not drug use, despite controlling for self-control and other covariates. In sum, a variety of studies show that the code of the street is interrelated with other psychological risk factors for crime and violence.

## 4. Current Focus

The current aim is to empirically examine whether the code of the street and its associations with delinquency, violence, and arrest can withstand confounds for psychological factors, namely psychopathy and temperamental deficits. Psychopathy and temperament similarly embody dispositional, interpersonal, and affective deficits that are conducive to aversive social interactions and antisocial behavior, and in this sense are conceptually congruent with the code of the street. In the event that psychopathy and temperament render the code of the street spurious lends credence to DeLisi’s suggestion that the code of the street is merely a sociological presentation of antisocial traits. In the event that the code of the street maintains significance with antisocial outcomes despite these controls provides considerable support.

## 5. Method

### 5.1. Participants and Procedures

We employed cross-sectional data derived from a sample of 253 juvenile offenders in long-term residential facilities in Pennsylvania. With Institutional Review Board (IRB) approval from the University of Pittsburgh, investigators collected data in 2009 and 2010 from a male-only facility (*n* = 152) and a female-only facility (*n* = 101). Researchers described the study to facility staff and youth, and a supervisor at each facility provided approval for the youth to participate in the study. Prior to administering the instrument, the interviewer explained the purpose of the study and received assent from minors and consent from those who were 18 or older. All interviewers completed intensive training and administered structured one-on-one interviews using computer-assisted survey interview (CASI) techniques. An interview editor was on-site to provide quality control for data collection procedures. Research staff conducted all interviews in private rooms and the CASI data collection procedure allowed the respondents to have each question read to them supplemented by response cards.

The current youth had extensive involvement in diverse forms of antisocial conduct and delinquency. Youth averaged 15 acts of delinquency, approximately 70% had sold drugs, and nearly 20% had their first juvenile court referral by age 12, which is suggestive of likely lifelong antisocial conduct [38]. In terms of behavioral history and risk factors for antisocial conduct, the current sample is comparable to other samples of serious delinquent youth in residential placement and related juvenile justice settings [39,40,41,42,43].

### 5.2. Measures

*Code of the Street.* We created a multiplicative term capturing a tendency toward irritability, anger, and aggressive responses to provocation and exposure to neighborhoods with low informal social control that is consistent with Anderson’s theory. The first component of the multiplicative term is the Angry–Irritable subscale derived from the Massachusetts Youth Screening Instrument Version 2 (MAYSI-2; [44]). It is a 9-item scale (0 = no, 1 = yes) designed to measure a tendency toward frustration, lasting anger, moodiness, and aggressive responses to interpersonal conflict (Cronbach’s α = 0.74). The items were exploratory factor analyzed and there was clear evidence of a single factor (Eigenvalue = 2.32). After varimax rotation, the factor loadings were “Have you lost your temper easily, or had a ‘short fuse?’” (λ = 0.52), “Have you been easily upset?” (λ = 0.49), “Have you thought a lot about getting back at someone you have been angry at?” (λ = 0.41), “Have you been really jumpy or hyper?” (λ = 0.23), “Have you had too many bad moods?” (λ = 0.58), “Have you felt angry a lot?” (λ = 0.64), “Have you gotten frustrated easily?” (λ = 0.62), “When you have been mad, have you stayed mad for a long time?” (λ = 0.35), and “Have you hurt or broken something on purpose, just because you were mad?” (λ = 0.27). The second component is a 4-item informal social control scale that measures the degree of prosocial monitoring and responsiveness in the youth’s rearing neighborhood (Cronbach’s α = 0.71) and the degree to which neighbors could be counted on to take action. Response categories involved a Likert scale where 1 = strongly agree, 2 = agree, 3 = neither agree nor disagree, 4 = disagree, and 5 = strongly disagree. The items were exploratory factor analyzed and there was clear evidence of a single factor (Eigenvalue = 1.39). After varimax rotation, the factor loadings were: “If children were skipping school and hanging out on a street corner” (λ = 0.62), “If children were spray painting graffiti on a local building” (λ = 0.52), “If children were ‘showing disrespect to an adult’” (λ = 0.62), and “If there was a fight in front of your house and someone was being beaten or threatened” (λ = 0.59). We set a cut point at the 50th percentile on the scale to capture those who lived in neighborhoods where adults generally would not intervene and exert informal social control and multiplied this binary term by the angry–irritable subscale. The resulting code of the street term (M = 2.84, SD = 3.26, range = 0–9) thus measured an aggressive/violent impulsive tendency to respond to interpersonal conflict among those reared in neighborhoods with little informal social control.

*Psychopathy.* The Youth Psychopathic Traits Inventory (YPI; [45]) is a self-report measure of psychopathy in adolescents (M = 105.74, SD = 20.92, range = 53–189). The YPI contains ten subscales that capture the various personality functioning and interpersonal style of psychopathy including dishonest charm, grandiosity, lying, manipulation, remorselessness, unemotionality, callousness, thrill-seeking, impulsiveness and irresponsibility. Prior research supports the YPI in terms of its reliability, convergent validity with other psychopathy measures and criterion validity with antisocial behaviors among delinquent and institutionalized youth [46,47,48,49,50].

*Temperament.* We measured temperament using DeLisi et al.’s [51] 15-item temperament scale (α = 0.88) reflecting deficits in effortful control and negative emotionality in accordance with DeLisi and Vaughn’s [52] temperament theory. Exemplar items include “Little things set me off,” “When I get really mad I hit someone,” “When I am angry I lose control over what I do,” “When I am angry I just lose it,” and “I am touchy and easily annoyed”. All items were exploratory factor analyzed and inspection of Eigenvalues showed clear evidence of a single factor (Eigenvalue = 5.20 with no other Eigenvalue factors > 1). Higher scores on the temperament measure indicate temperamental features characterized by low effortful control and/or greater negative emotionality, which research has shown to have significant associations with antisocial behavior [53,54,55,56].

### 5.3. Demographic Covariates

We controlled for sex (female = 0, 39.7%; male = 1, 60.3%), African American (no = 0, 48%; yes = 1, 52%), and age (M = 15.98, SD = 1.42, range = 13–19).

### 5.4. Dependent Variables

We utilized three self-reported dependent variables. *Self-reported delinquency* (M = 15.29, SD = 13.16, range = 0–65) and *self-reported violent delinquency* (M = 8.94, SD = 7.84, range = 0–35) were based on the self-report of delinquency employed in the National Youth Survey Family Study [57]. This is among the most widely used self-report delinquency measures and it has convergent validity with official measures of crime [58]. The delinquency items included motor vehicle theft; theft over $50; bought or sold stolen goods; stolen marijuana or other drugs; carrying a hidden weapon; gang fighting; hitting a teacher; hitting a parent; hitting other students; strong arming students, parents, and teachers; hitting an animal; and attacking someone. *Self-reported arrests* (M = 3.73, SD = 4.0, range = 0–32) was the number of police contacts the youth experienced prior to residential placement.

### 5.5. Analysis

To test whether the association between the code of the street and antisocial behavior withstood competing confounds, we specified hierarchical negative binomial regression models with incidence rate ratios for self-reported arrests, delinquency, and violence. Negative binomial regression is appropriate to estimate count data dependent variables [59,60], such as self-reported delinquency and arrests, when there is overdispersion where the variance exceeds the mean. The likelihood ratio test of α was conducted and reported in each table, confirming the negative binomial and not the Poisson estimator was appropriate. In model 1, the code of the street was the only independent variable. In model 2, we specified the demographic controls, and in model 3 psychopathy and temperament were included. We conducted sensitivity analyses by examining the models by African American status given its salience to street code theory. To increase confidence in the estimates, we specified bootstrapped standard errors with 500 replications. Finally, in order to allow for comparison of fit across models, we specified the estat ic command in Stata 14.2 [61] to produce Akaike information criterion (AIC) and Bayesian information criterion (BIC) statistics, where lower values indicate better model fit.

## 6. Findings

### 6.1. Negative Binomial Regression Models for Self-Reported Arrests

As shown in Table 1, the code of the street had a significant association with self-reported arrests in the baseline model (IRR = 1.05, z = 2.46, *p* < 0.05). The specification of age, African American, and sex in model 2 did not mitigate the code of the street as its association remained significant (IRR = 1.06, z = 2.57, *p* < 0.01). In the fully specified model, the code of the street retained significance (IRR = 1.05, z = 2.43, *p* < 0.05) as youth with greater advocacy of the street code reported more arrests. Males and youth with more psychopathic features also reported more arrests during their delinquent career.

### 6.2. Negative Binomial Regression Models for Self-Reported Delinquency

As shown in Table 2, the code of the street had a significant association with self-reported delinquency in the baseline model (IRR = 1.06, z = 2.79, *p* < 0.01). The specification of age, African American, and sex in model 2 did not mitigate the code of the street as its association remained significant (IRR = 1.06, z = 3.18, *p* < 0.001). In the fully specified model, the code of the street retained significance (IRR = 1.04, z = 2.18, *p* < 0.05) as youth with greater advocacy of the street code reported more delinquent involvement. Youth with more psychopathic features and temperamental features characterized by negative emotionality and low self-regulation reported more delinquency.

### 6.3. Negative Binomial Regression Models for Self-Reported Violence

As shown in Table 3, the code of the street had a significant association with self-reported violence in the baseline model (IRR = 1.04, z = 2.70, *p* < 0.01). The specification of age, African American, and sex in model 2 did not mitigate the code of the street as its association remained significant (IRR = 1.05, z = 2.82, *p* < 0.01). In the fully specified model, the code of the street retained significance (IRR = 1.03, z = 2.11, *p* < 0.05) as youth with greater advocacy of the street code reported more violent delinquent acts. Males and youth with negative, poorly regulated temperaments also reported more violence.

### 6.4. Negative Binomial Regression Models for Self-Reported Arrests by African American Status

As shown in Table 4, we specified the models split by African American status. For non-African Americans, the code of the street had a null association with self-reported arrests. Males and those with greater psychopathy also reported more arrests. Among African Americans, the code of the street was significantly associated with self-reported arrests (IRR = 1.06, z = 2.92, *p* < 0.01). Among African Americans, age was inversely associated with self-reported arrests.

### 6.5. Negative Binomial Regression Models for Self-Reported Delinquency by African American Status

As shown in Table 5, we specified the models split by African American status. For non-African Americans, the code of the street had a null association with self-reported delinquency. Among non-African Americans, youth with greater psychopathy and more difficult temperaments also reported more delinquency. Among African Americans, the code of the street was significantly associated with self-reported delinquency (IRR = 1.04, z = 2.37, *p* < 0.05). Among African Americans, psychopathy and temperament were also positively associated with delinquency.

### 6.6. Negative Binomial Regression Models for Self-Reported Violence by African American Status

As shown in Table 6, we again specified the models split by African American status. For non-African Americans, the code of the street had a null association with self-reported violence. Among non-African Americans, males and youth with more difficult temperaments also reported more violence. Among African Americans, the code of the street also had a null association with self-reported violence. Among African Americans, males and youth with more difficult temperamental features were also positively associated with violence.

## 7. Discussion

The trend in African American offending rates being as much as nine times higher than white offending rates in the same or similar environments is a significant issue that deserves and requires explanation from criminologists. A large number of studies have utilized macro-level theories to explain the sociological, cultural, and structural reasons for such discrepancies in offending patterns, yet considerably less attention has been directed toward exploring the effect of individual personality deficits about offending in such environments. Scholars have speculated [18,19,20,21] that Anderson’s code of the street was merely a sociological presentation of antisocial traits. The current study represents one such investigation as we empirically examined whether the code of the street and its association with delinquency, arrests, and violence can withstand confounds for psychological personality traits, specifically psychopath and temperament deficits, using a sample of institutionalized juvenile offenders in a long-term residential facility. Results demonstrated that the code of the street fared well, suggesting that there are in fact structural and cultural features to the code that are beyond individual personality traits. Several findings warrant discussion.

First, the consistent linkages between the code of the street and the outcome variables herein are impressive given the quality and nature of the psychopathy and temperament variables. As they instantiate the essential features of an individual with self-regulation and conduct problems, both psychopathy and temperament exist in general theories of antisocial behavior in the social sciences [62,63,64,65,66,67,68]. Particularly in the case of DeLisi and Vaughn’s theory that presents an individual with core self-regulation deficits and abundant negative emotionality, it is impressive that the code of the street maintained predictive validity in the models, given that similar explanatory variance typified the psychological measures. Consistent with prior research [32], this suggests that it is not just anger dysregulation, vengefulness, or mere temper that explains the association between street code adherence and antisocial conduct, but there are emergent sociological features to the street code beyond the contributions of psychopathy and temperament. Perhaps the code of the street is not simply a form of self-presentation, but acts as a cultural narrative actors can use to build their identity and orient their behaviors [16]. For some, criminal offending is a choice driven by the need to maintain the street persona that is their identity.

Second, Anderson’s [1,2] theory is unique in that it attempted to explain violent and antisocial conduct uniquely among African Americans. Although there is evidence that code of the street values transcend race, ethnicity, and nationality [69,70], the current models show that in addition to the main effects, the code of the street has null associations with offending among non-African Americans but significant linkages with self-reported arrests and self-reported delinquency among African Americans. Curiously, the split race models also showed that the code of the street was not associated with self-reported violence among African Americans, despite that being a central postulate of the theory. One explanation for this unexpected finding is the violent delinquency measure does not contain homicide, which is the quintessential example of a violent interaction where lethality is used to save face or respond to disrespect. Prior researchers have referred to these lethal encounters as “cultural retaliatory homicide” [71]. Alternatively, our operationalization of street code may capture individuals who are “code switchers” and vacillate in adherence to the street code per the situation, but may be less likely to act violently than those who fully embodied the code of the street. Even among youth, stronger and long-term adherence to the code of the street is associated with increased violent offending [35]. Nevertheless, the code of the street was more salient in models of delinquency and arrest among African Americans than other youth, which is consistent with prior research [72].

Third, perhaps because Anderson’s theory involves a qualitative approach, criminologists have devised a variety of measures to operationalize the code of the street and no single measure has achieved consensus in the literature. The current measure combined the angry, aggressive, aggrieved disposition with exposure to neighborhoods where deviance is tolerated and unlikely to be sanctioned by neighbors. We strongly encourage researchers to explore connections between the code of the street and other psychological constructs related to antisocial behavior. For instance, in the current data, the code of the street had small yet significant correlations with several subscales of the YPI including manipulation (r = 0.15, *p* < 0.02), thrill-seeking (r = 0.15, *p* < 0.01), impulsiveness (r = 0.14, *p* < 0.03), and irresponsibility (r = 0.16, *p* < 0.01). Although the code of the street withstood competing effects for psychopathy, it is also clear that psychopathic features partially imbue the street code.

Fourth, although the primary research purpose was theory testing, there is also practical value to our findings. Across models, it was clear that seriously delinquent youth exhibit a variety of antisocial features relating to their temperament, their personality functioning, and subcultural adaptations like the street code. Of these three antisocial features, there is frankly greater likelihood for change for the street code, since temperament and personality are moderately to highly heritable and mostly stable across life, especially among those who would meet diagnostic criteria for personality and conduct disorders (e.g., Conduct Disorder, Antisocial Personality Disorder, Intermittent Explosive Disorder). In contrast, the code of the street is reducible in multiple ways, including improving police–community relations so that street code adherents are empowered to employ lawful responses to disputes as suggested by prior researchers [73]. Additionally, school-based crime and violence prevention programing, such as the G.R.E.A.T. program, which aims to foster prosocial conflict resolution and cooperation skills shown to reduce gang membership and violence while improving prosocial outcomes [74,75].

Another approach involves correctional interventions, such as Aggression Replacement Training (ART; [76,77]. ART treats aggression and violence as a multifaceted construct that has behavioral, affective, and moral features and the intervention involves the inculcation of prosocial responses to conflict, anger control and management, and moral reasoning. These precepts map well to the code of the street in terms of helping youth recognize the alternatives to a violent (or potentially homicidal) encounter, helping the youth in their maintenance of anger but also maintenance of excessive feelings of pride that often serve as the motivator to avenge disrespect. Although it is not a gold-standard intervention (for a recent systematic review, see, [78], ART does have effectiveness among offender and adolescent at-risk populations [79,80,81]. In summary, our operationalization and findings translate into evidence informed actionable interventions.

Findings from the present study should be interpreted in the context of several limitations. First, given the cross-sectional nature of the study, temporal ordering cannot be established, which limits the ability to draw causal inferences. Moreover, the study variables were based on youth self-reports, making responses subject to social desirability bias. For instance, some youth may be more inclined to report about favorable traits to researchers. Additionally, though the study sample was demographically comparable to national samples of juvenile offenders, the geographic context of the study sample (Eastern United States) may makes comparisons to other regions of the country difficult. Moreover, neighborhood conditions were based entirely on self-perceptions and we had no objective assessments of neighborhood characteristics to draw from.

## 8. Conclusions

Despite these limitations, the present study offers a fresh perspective about the code of the street conceptualization. Our results indicate the code of the street remained a significant factor for predicting antisocial behavior in the face of well-known psychological constructs, such as psychopathy and temperamental traits and other controls. Consistent with theory, the code of the street factor was uniquely influential for African American youth while general temperament difficulties were more salient for non-African American youth compared to African American youth. Importantly, the code of the street is likely more malleable to evidence-informed steps than core psychopathy and temperament traits.

## Figures and Tables

**Table 1:** Negative binomial regression models for self-reported arrests.

Variable	IRR (BSE)	z	IRR (BSE)	z	IRR (BSE)	z
Code of the Street	1.05 (0.02)	2.46 *	1.06 (0.02)	2.57 **	1.05 (0.02)	2.43 *
Age			0.96 (.04)	−1.02	0.95 (0.04)	−1.11
African American			0.99 (.12)	−0.06	0.98 (0.10)	−0.18
Sex			1.36 (.20)	2.08*	1.34 (0.14)	2.79 **
Psychopathy					1.01 (0.01)	2.75 **
Temperament					1.01 (0.01)	1.08
Wald χ2	7.89 **		13.39 **		36.83 ***	
LR Test of α	224.26 ***		207.64 ***		190.75 ***	
AIC	1067.70		1064.94		1051.37	
BIC	1077.93		1085.41		1078.62	

*** *p* < 0.001, ** *p* < 0.01, * *p* < 0.05.

**Table 2:** Negative binomial regression models for self-reported delinquency.

Variable	IRR (BSE)	z	IRR (BSE)	z	IRR (BSE)	z
Code of the Street	1.06 (0.02)	2.79 **	1.06 (0.02)	3.18 ***	1.04 (0.02)	2.18 *
Age			0.99 (0.04)	−0.22	1.00 (0.03)	0.11
African American			0.97 (0.11)	−0.25	1.02 (0.11)	0.15
Sex			1.05 (0.12)	0.44	1.17 (0.13)	1.41
Psychopathy					1.01 (0.01)	3.99 ***
Temperament					1.04 (0.01)	4.63 ***
Wald χ2	7.80 **		10.56 *		89.7 ***	
LR Test of α	1563.45 ***		1560.55 ***		1159.59 ***	
AIC	1677.23		1682.98		1626.75	
BIC	1687.47		1703.45		1654.01	

*** *p* < 0.001, ** *p* < 0.01, * *p* < 0.05.

**Table 3:** Negative binomial regression models for self-reported violence.

Variable	IRR (BSE)	z	IRR (BSE)	z	IRR (BSE)	z
Code of the Street	1.04 (0.02)	2.70 **	1.05 (0.02)	2.82 **	1.03 (0.01)	2.11 *
Age			0.98 (0.03)	−0.43	1.01 (0.04)	0.36
African American			0.95 (0.09)	−0.51	0.95 (0.11)	−0.46
Sex			1.30 (0.19)	1.78	1.52 (0.17)	3.63 ***
Psychopathy					1.01 (0.01)	1.73
Temperament					1.05 (0.01)	5.99 ***
Wald χ2	7.31 **		9.08		144.03 ***	
LR Test of α	864.37 ***		837.47 ***		596.21 ***	
AIC	1453.58		1455.48		1406.81	
BIC	1463.82		1475.95		1434.06	

*** *p* < 0.001, ** *p* < 0.01, * *p* < 0.05.

**Table 4:** Negative binomial regression models for self-reported arrests by African American status.

	Non-African American		African American	
Variable	IRR (BSE)	z	IRR (BSE)	z
Code of the Street	1.03(0.04)	0.86	1.06 (0.02)	2.92 **
Age	1.04 (0.08)	0.55	0.86 (0.06)	−2.37 *
Sex	1.43 (0.26)	1.98 *	1.20 (18)	1.24
Psychopathy	1.01 (0.00)	2.62 **	1.0 (0.01)	0.54
Temperament	1.01 (0.01)	0.79	1.02 (0.01)	1.32
Wald χ2	23.7 ***		24.59***	
LR Test of α	84.6 ***		91.79***	
AIC	510.45		544.39	
BIC	529.29		563.54	

*** *p* < 0.001, ** *p* < 0.01, * *p* < 0.05.

**Table 5:** Negative binomial regression models for self-reported delinquency by African American status.

	Non-African American		African American	
Variable	IRR (BSE)	z	IRR (BSE)	z
Code of the Street	1.02 (0.03)	0.79	1.04 (0.02)	2.37 *
Age	0.96 (0.06)	−0.62	1.01 (0.06)	0.14
Sex	1.30 (0.20)	1.67	1.13 (0.17)	0.79
Psychopathy	1.01 (0.01)	2.73 **	1.01 (0.00)	2.46 *
Temperament	1.06 (0.01)	5.13 ***	1.03 (0.01)	2.08 *
Wald χ2	74.2 ***		32.5 ***	
LR Test of α	568.18 ***		550.21 ***	
AIC	783.68		847.99	
BIC	802.52		867.15	

*** *p* < 0.001, ** *p* < 0.01, * *p* < 0.05.

**Table 6:** Negative binomial regression models for self-reported violence by African American status.

Variable	IRR (BSE)	z	IRR (BSE)	z
Code of the Street	1.04 (0.03)	1.30	1.01 (0.02)	0.60
Age	0.95 (0.05)	−0.99	1.07 (0.06)	1.17
Sex	1.48 (0.26)	2.21 *	1.66 (0.28)	2.98 **
Psychopathy	1.01 (0.01)	1.49	1.0 (0.01)	0.81
Temperament	1.06 (0.01)	4.31 ***	1.04 (0.01)	3.58 ***
Wald χ2	67.49 ***		81.04 ***	
LR Test of α	321.74 ***		255.86 ***	
AIC	689.32		724.85	
BIC	708.16		744.01	

*** *p* < 0.001, ** *p* < 0.01, * *p* < 0.05.
